# Knowledge and Perception of Orthodontic Treatment among General and Non-Orthodontic Dental Specialists: A Comparative Study

**DOI:** 10.3390/healthcare11030340

**Published:** 2023-01-24

**Authors:** Abdulrahman Khulaif Alshammari, Abeer Alanazi, Haya Al-swedani, Mahvish Khan, Saheem Ahmad, Shafiul Haque, Saif Khan

**Affiliations:** 1 Preventive Department, College of Dentistry, Ha’il University, Ha’il 2440, Saudi Arabia; 2 Ministry of Health, Ha’il 2440, Saudi Arabia; 3 Private Clinic, Ha’il 2440, Saudi Arabia; 4 Department of Biology, College of Science, Ha’il University, Ha’il 2440, Saudi Arabia; 5 Department of Clinical Laboratory Sciences, College of Applied Medical Sciences, Ha’il University, Ha’il 2440, Saudi Arabia; 6 Research and Scientific Studies Unit, College of Nursing & Allied Health Sciences, Jazan University, Jazan 45142, Saudi Arabia; 7 Basic Dental and Medical Sciences Department, College of Dentistry, Ha’il University, Ha’il 2440, Saudi Arabia

**Keywords:** orthodontic knowledge, orthodontic attitude, orthodontic awareness, orthodontic perception, orthodontic treatment

## Abstract

The objective of this study is to discover any possible association of gender or practitioner classification with orthodontic knowledge and attitudes towards orthodontic treatment. Descriptive cross-sectional research was conducted on two groups of dentists in the Ha’il region of Saudi Arabia. Group I included general practitioners, while Group II had non-orthodontic specialists. The findings suggest a statistically significant (*p* < 0.05) difference between the knowledge and attitudes of general practitioners and non-orthodontic specialists. Independent two tailed t-scores for difference in Knowledge (t-score = 3.19919, *p* = 0.003) and Attitude (t-score = 2.16314, *p* = 0.048319), highlight significant disparities in the knowledge and attitudes of general practitioners and non-orthodontic specialists. However, no statistically significant difference was observed in terms of knowledge and attitudes based on gender differences. This study captures and highlights subtle information that is very significant in dealing with critically important orthodontics issues. The study suggests that it is possible that a non-orthodontic specialist may suggest an altogether different line of treatment with different consequences when compared to a general practitioner and vice versa. This may result in unwanted, permanent orthodontic effects, highlighting the significance of the early stage of orthodontic treatment awareness. This research reveals disparities between the perceptions of general practitioners and non-orthodontic specialists about the stage and relevance of orthodontic treatment. It is strongly advised to see an orthodontic expert rather than a general practitioner or non-orthodontic specialist for orthodontic issues.

## 1. Introduction

Oral health is very important for each individual, and it affects the general health of the body. In several cultures, parents and adolescents are unaware of the causes, prevalence, and prevention of oral disorders [1]. One of the frequent development anomalies of the oral cavity is malocclusion. Malocclusion is considered one of the causes of caries development and periodontal disease [2]. Malocclusion also reduces masticatory function and efficiency [3,4]. Impaired chewing function affects nutrition and the overall health status of people [5,6,7,8,9]. Malocclusion is described as discrepancies between the jaws, including an abnormal relationship between the maxillary and mandibular dental arches or anomalies within the jaws [10]. Malocclusion generally bring a feeling of disfavor about facial appearance and tentative feelings in society [11]. The etiology of malocclusion can be heredity or environmental and/or a combination of both along with various local factors such as thumb sucking and tooth irregularities [12].

Orthodontics is a branch of dentistry that focuses on correcting malocclusions of the mandible, maxilla, and teeth. The benefit of orthodontic therapy is the avoidance of tissue damage, both aesthetically and in terms of somatic function [2]. The other critical points of interest are the upgrading of individual fulfillment; the enhancement of confidence; and bodily, psychical, and sociable enhancement. It is essential to teach people about the advantages of having normal occlusion and the significance of impediments and orthodontic treatment [13]. Non-orthodontic specialists and general dentists ought to have the information of the essential standards of orthodontics to teach patients, to analyze their issues accurately, and for appropriate referral. This is accomplished by merging numerous professional specialties in a way that allows general practitioners and non-orthodontic specialists to take the role of oral health educators if they have excellent awareness and understanding of orthodontic treatment standards and attitudes [13,14,15,16,17,18,19]. To improve patient orthodontic care, it is vital to determine the degree of orthodontic treatment knowledge among general practitioners and non-orthodontic specialists. The stage of orthodontic intervention is critical for the achievement of the desired benefits [20,21]. A non-standard orthodontic intervention of a well-defined orthodontic issue may result in permanent orthodontic deformities [22,23].

Statistically, this study determines which of the following hypotheses in terms of practitioner classification or gender are acceptable or significant.

### 1.1. Practitioner Classifications Hypothesis

Null hypothesis: There are no statistically significant differences between the two categories of practitioners (general and non-orthodontic specialists) with respect to total knowledge and attitude.

Alternative hypothesis: There are statistically significant differences between the two categories of practitioners (general and non-orthodontic specialists) with respect to total knowledge and attitude.

### 1.2. Gender Difference Hypothesis

Null hypothesis: There are no statistically significant differences between the two genders (male and female) with respect to total knowledge and attitude.

Alternative hypothesis: There are statistically significant differences between the two genders (male and female) with respect to total knowledge and attitude.

The purpose of this study is to compare the awareness and the attention about standards of orthodontic treatment in terms of stage of intervention and relevance among dentists and practicing dental specialists other than the orthodontist.

## 2. Materials and Methods

### 2.1. Sample Size and Questionnaire Design

A descriptive cross-sectional study was conducted on 48 dentists. They were chosen from different area of Ha’il, Saudi Arabia, to study the attitudes and awareness of the basics of orthodontic therapy. The distribution of the study was conducted through online surveys. The duration of the study was five months, starting on 28 October 2019. The answers to the questions were either yes or no. For both knowledge and attitude, the yes answers were coded by one, while the no answers coded by zero. The inclusion and exclusion criteria are given in Table 1.

**Figure 1:**
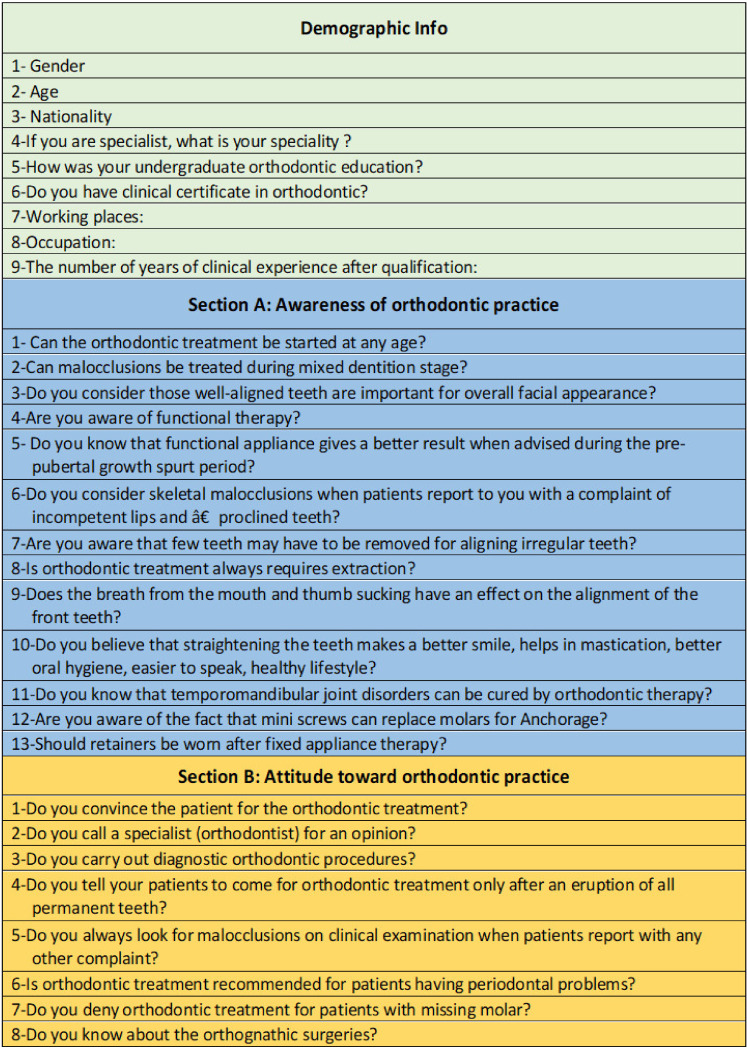
Sample questionnaire.

The study was conducted by a questionnaire containing 21 questions (Figure 1) divided into two sections: section A contained 13 questions about awareness of orthodontic practice, and section B comprised 8 questions about attitudes toward orthodontic practice taken from a previous study^1^. The questionnaire was planned to study the attitudes and awareness towards the basics of orthodontic therapy of the general dental practitioners and non-orthodontic specialists. The internal consistency of the questionnaire was also tested via Cronbach’s alpha test on the SPSS v. 25.

Section A: Survey for the awareness of orthodontic practice:

In total, 13 queries were defined to gauge the information on general dental practitioner and non-orthodontics specialists. The inquiries were comprised of their insights with respect to facial appearance, mixed orthodontic treatment in mixed dentition organization, the beginning period of orthodontic treatment, practical treatment, habits, extraction of teeth for orthodontic reasons, proclined teeth, retainers, and anchorage. For scoring, the yes answer was given a score of “one”, and “zero” was offered for the response of no.

Section B: Survey to consider attitudes toward orthodontic practice:

These 8 questions were likewise designed with a yes/no pattern, and scoring was comprised of a score of “one” for a yes answer and a score of “zero” for a no answer. In these questions, the plan was to examine attitudes toward orthodontic treatment, such as offering data tolerant about malocclusion when patient seeks the other dental treatment, assessment of orthodontists, diagnostic orthodontic techniques, orthodontic treatment in missing teeth, orthognathic surgical procedures, and orthodontic therapy in patients with periodontal issues.

The Cronbach alpha value for the 13 awareness questions was “0.9” and for the 8 attitude 8 questions was “0.83”, which fell into the acceptable category.

The survey was distributed randomly to general dental practitioners and non-orthodontic specialists through individual online surveys and through personal contacts, phone, and email.

### 2.2. Statistical Analysis

The data analysis of the current study was conducted using descriptive and inferential statistical tests. Mean, standard deviation, and standard of error were used as descriptive statistics. Moreover, for inferential statistics, independent *t*-tests were used to meet the specific objectives. The level of significance was set at α = 0.05, and all hypothesis testing was conducted using two-sided tailed hypotheses. In addition, the statistical program utilized was the IBM SPSS 25 Statistical Package for the Social Sciences.

## 3. Results

### Respondents’ Characteristics

Data were collected from 48 respondents from the target population of GPs and specialists. However, gender, age, nationality, specialty, undergraduate knowledge, working place, and occupational years of experiences were the demographic variables collected from respondents. Counts and percentages of each category of each variable are presented in Table 2, which shows detailed information about the respondents’ characteristics. Table 3 shows the distribution between gender and respondents’ specialties. The results indicated that females had higher associations than males in terms of general practitioners or non-orthodontic respondents.

In term of knowledge and attitudes between general dental practitioners and non-orthodontic practitioners, descriptive statistics are shown in Table 4.

To determine whether there is a significant difference in knowledge and attitudes between the two genders, independent *t*-tests were conducted. The following hypotheses were considered for the *t*-tests:

Null hypothesis: There are no statistically significant differences between the two genders (male and female) with respect to total knowledge and attitudes.

Alternative hypothesis: There are statistically significant differences between the two genders (male and female) with respect to total knowledge and attitudes.

The results indicated that we rejected the alternative hypothesis and accepted the null hypothesis. There are no significant differences between the two genders in terms of knowledge or attitudes. Table 5 summarizes the results of the independent *t*-test.

To determine the differences in knowledge and attitudes between the two categories of practitioners, namely, general and non-orthodontic specialist, independent *t*-tests were conducted. The following hypotheses were considered for the *t*-tests:

Null hypothesis: There are no statistically significant differences between the two categories of practitioners (general and non-orthodontic specialists) with respect to total knowledge and attitudes.

Alternative hypothesis: There are statistically significant differences between the two categories of practitioners (general and non-orthodontic specialists) with respect to total knowledge and attitudes.

The results indicated that we accepted the alternative hypothesis and rejected the null hypotheses. There is a significant difference in the knowledge and attitude of the general practitioner when compared to non-orthodontic specialists irrespective of gender. Table 6 shows the results of independent *t*-tests.

## 4. Discussion

In this study, a relative assessment was accomplished to assess the information and attention of general dental practitioners and non-orthodontic specialists with the guide of prepared questionnaires. At the point when the examination of the information scores between general dental practitioners and non-orthodontic specialist was done, it demonstrated profoundly critical contrasts. The information of the dental specialists was shown, who experienced the preparation of three additional years after their graduation, which was more when compared in relation to general dental practitioners [14]. When all the members were given the information question of the beginning of the orthodontic treatment at any age, 64.6% of them answered yes. The treatment of malocclusions during the mixed dentition stage and the importance of well-aligned teeth for the overall facial appearance were answered affirmatively by 87.5% and 93.8%, respectively. About an 83.3% positive response resulted for the awareness of extraction of a few teeth for the aligning of irregular teeth in the case of non-orthodontic specialists and 91.3% for general dental practitioners. The maximum positive response, i.e., 86%, was given by general dental practitioners for the effects of habits such as mouth breathing or thumb sucking on alignment of the front teeth. Yes answers to the inquiry of the utilization of retainers and mini screws were 91.7% and 62.5% by the general dental experts and 87.5% and 87.5% by the non-orthodontic specialists, respectively; i.e., 87.5% of non-orthodontic specialists knew about how mini screws can substitute molars for anchorage, while 12.5% were inexperienced with the idea. The comparison of the attitudes toward orthodontic referral and practice between general dental experts and non-orthodontic specialists showed slight differences. In the investigation of attitude questions, the greatest affirmative reaction (100%) in general dental experts was given for calling an orthodontist for an opinion, and that for non-orthodontic specialists (91.7%) was given for knowledge about orthognathic procedures. Examination of a malocclusion on clinical assessment when a patient reports with some other problems was answered “yes” by 79.5% of non-orthodontic specialists and 71% of dentist. Among all members, around 29% accepted that orthodontic treatment can be done in patients with periodontal issues, while 71% did not. In total, 29% of dentists disbelieved the need for orthodontic treatment for patients with missing molars. At the point when the general scores of male and female specialists were examined, male members displayed higher scores when contrasted with females; however this difference was not statistically significant. This indicated that male dental professionals do not possess significantly more positive information and attitudes than female dental specialists toward the practices and principals of orthodontic treatment. A significant difference in the knowledge and attitudes of general dental practitioners was observed when compared to non-orthodontic specialists, indicating that both general dental practitioners and specialists should and must become involved in the continuous updating of their understanding of dental specialties, irrespective of their specific specialty. Social media and other public access platforms may play an important role in enhancing the knowledge and awareness regarding orthodontic treatment among general practitioners and non-orthodontic specialists, as well as the patients [24,25]. A comparative study of social media-based instructions for orthodontic issues from orthodontists, general practitioners, and non-orthodontic specialists may provide interesting information. This information may be implemented to devise continuous study programs and, if required, modify the basic dental curriculum to better equip our future dentists.

It is possible that the general practitioner or a non-orthodontic expert may suggest an overall distinct treatment strategy for critical orthodontic issues with alternative outcomes.

## 5. Conclusions

The current investigation showed the necessity for extended clinically arranged preparations with respect to orthodontic treatment. Thus, the schedules for undergraduate programs should include remedial thoughts and promote continuous education for general dental practitioners to overhaul their knowledge of orthodontic case management. General practitioners as well as non-orthodontic specialists should be equipped with the ability to identify bad orientations of orthodontic treatments at the early stages of orthodontic issues and advise patients to obtain a thorough orthodontic examination by the field specialist only. It is strongly advised that general practitioners or non-orthodontic experts should advise patients to visit orthodontic experts for critical orthodontic issues.

## Figures and Tables

**Table 1:** Inclusion and exclusion criteria.

Inclusion Criteria	Exclusion Criteria
A general dental practitioner with a bachelor’s degree.	Dentists more than 50 years old.
Dentist with post-graduation degree in any branch of non-orthodontic specialty.	General practitioners who stopped their practice.
Age between 25 and 50 years.	Orthodontic specialists.

**Table 2:** Respondents’ characteristics (*n* = 48).

Variables	Categories	Frequency	%
Gender	Male	20	41.7
	Female	28	58.3
Nationality	Non-Saudi	20	41.7
	Saudi	28	58.3
Specialties	GP	25	52.1
	Dental Public Health	3	6.3
	Endodontic	4	8.3
	Oral and Maxillofacial Surgery	2	4.2
	Oral and Maxillofacial Radiology	1	2.1
	Periodontics	4	8.3
	Prosthodontics	4	8.3
	Restorative	1	2.1
	Pediatric Dentistry	4	8.3
How was your undergraduate orthodontic education?	Poor	3	6.3
	Fair	5	10.4
	Good	26	54.2
	Very Good	10	20.8
	Excellent	4	8.3
Occupation	General Practitioner	23	47.9
	Specialist	22	45.8
	Consultant	3	6.3
Experience	Less than 1 year	6	12.5
	1–5 years	19	39.6
	6–10 years	10	20.8
	More than 10 years	13	27.1

**Table 3:** Comparison between gender and specialty (*n* = 48).

Variables	Categories	Gender		Total
		Male	Female	
Specialty	General dental practitioner	11	14	25
		44.0%	56.0%	100.0%
	Non-orthodontic specialties	9	14	23
		39.1%	60.9%	100.0%
Total		20	28	48
		41.7%	58.3%	100.0%

**Table 4:** Comparison of knowledge and attitudes between practitioner classification and gender (*n* = 48).

Variables	Specialty	Gender	*n*	Mean/*n*	SD
Total knowledge score	General dental practitioner	Male	7	0.791	0.181
		Female	16	0.899	0.129
		Total	23	0.866	0.117
	Non-orthodontic specialty	Male	13	0.680	0.241
		Female	12	0.654	0.163
		Total	25	0.668	0.191
Total attitude score	General dental practitioner	Male	7	0.768	0.170
		Female	16	0.641	0.306
		Total	23	0.679	0.209
	Non-orthodontic specialty	Male	13	0.490	0.184
		Female	12	0.469	0.166
		Total	25	0.480	0.156

**Table 5:** Comparison of knowledge and attitudes among practitioners’ genders (*n* = 48).

Variables	Gender	*n*	Mean/*n*	SD	t	df	*p*
Total knowledge	Male	20	0.736	0.169	0.674	46	0.506
	Female	28	0.776	0.135			
Total attitude	Male	20	0.629	0.111	−0.84	46	0.413
	Female	28	0.555	0.223			

**Table 6:** Comparison of knowledge and attitude among practitioners classification (*n* = 48).

**Variables**	**Specialty**	***n***	**Mean/*n***	**SD**	**t**	**df**	***p***
Total knowledge	General dental practitioner	25	0.866	0.117	3.199	46	0.003
	Non-orthodontic specialties	23	0.668	0.191			
Total attitude	General dental practitioner	25	0.679	0.209	2.163	46	0.048
	Non-orthodontic specialties	23	0.480	0.156			

## Data Availability

Not applicable.
